# Association between Maternal Mortality and Cesarean Section: Turkey Experience

**DOI:** 10.1371/journal.pone.0166622

**Published:** 2016-11-23

**Authors:** Cihangir Uzuncakmak, Hasene Ozcam

**Affiliations:** 1 Fatih Sultan Mehmet Training and Research Hospital Obstetrics and Gynecology Department, Fatih Sultan Mehmet Hastanesi E5 Karayolu Uzeri Icerenkoy Atasehir,34752, Istanbul, Turkey; 2 Istanbul Training and Research Hospital Obstetrics and Gynecology Department, Kasap Ilyas Mah. Org. Abdurrahman Nafiz Gurman Cd., 34098, Istanbul, Turkey; Stellenbosch University, SOUTH AFRICA

## Abstract

**Background:**

To investigate the cesarean Section (C/S) rates and maternal mortality (MM) causes and its relation between 2002 and 2013.

**Methods:**

Data were gathered from Turkish Ministry of Health and Istanbul Health Administration. The Annual Clinical Reports for 2002–2013 were reviewed and analyzed: C/Ss and maternal deaths in women who gave birth ≥20 weeks between January 1, 2002, and December 31, 2013, in any hospital in Turkey and Istanbul.

**Results:**

The major causes of MM were hemorrhage (20%), hypertensive disorders (18.2%), embolism (10.3%), cardiovascular conditions (9%), infection (8.5%), and other causes (10.4%). Overall, the average annual CS delivery rate was 46.4% in Istanbul and 36.6% in Turkey. There was a significant increase in the CS rates in Istanbul and Turkey from 2008 to 2013 relative to those from 2002 to 2007 (p = 0.004). There was a statistically significant and inverse relationship (97.2%) between the MMR and CS rate from 2002 to 2013 in Turkey (p = 0.001). However, no significant relationship was detected between the MMR and CS rate from 2002 to 2013 in Istanbul (p > 0.05). There was a significant inverse correlation (66.3%) between the CS rate and peripartumhemorrhage in Turkey (p = 0.019) and there was a significant inverse correlation (66.5%) between the CS rate and peripartumhemorrhage(p = 0.018) in Istanbul between 2007 to 2013. There were no significant differences in ante-intrapartum haemorrhage bleeding (p > 0.05) or postpartum hemorrhage (p > 0.05) from 2007 to 2013.

**Conclusions:**

This study demonstrates that there was a inverse correlation between increased CS and maternal mortality rates during the previous decade in Turkey. Although cesarean rates increase excessively, it appears that improved health care facilities have a positive effect on MMRs in Turkey.

## Introduction

Cesarean section (CS) is a major surgical procedure that can save the lives of both fetuses and mothers. However, a medically inappropriate CS is associated with a higher risk of perinatal and maternal mortality than is a vaginal delivery [1]. Studies have found that most cases of obstetric hemorrhage and emergency postpartum hysterectomy were associated with previousCSdeliveries [2]. Due to the surgical nature of CS, after controlling for indication bias and confounders, the risk of postpartummaternaldeath is almost three-fold higher than that associated with vaginal delivery; these maternal deaths are mainly due to postpartum hemorrhage and complications of anesthesia. CS delivery is an independent risk factor for postpartummaternaldeath [3].

Nevertheless, access to CS is essential for maternal and fetal health, reducing the maternal mortality ratio (MMR) and decreasing disability. In countries with an inadequate surgical capacity and limited access to CS, it has been estimated that increased access to emergency and urgent CS may decrease the MMR by as much as 92% [4]. A previous study showed that target CS rates can be achieved by identifying the common indications for CS and their associations withmortality,thus improving antenatal services and emergency obstetric care [5].

The major cause of maternal death in developed countries is thromboembolism, whereas obstetric hemorrhage is the most common cause of maternal death in low-and middle income countries [6].

MMRs in low-income countries (LICs) are unacceptably high despite efforts taken to reduce MMRs through the Millennium Development Goal 5 of the World Health Organization (WHO) [7]. Despite progress, inequality prevails both among and within countries.

Among all European cities, Istanbul has the largest population (>14 million citizens) and a 1.8% birth rate. CS delivery rates in Turkey are at an all-time high; the current rate of 49% is approximately six-fold higher than that during the 1990s. The rate of CS in Istanbul increases to >55% when hospital-registered deliveries are also included [8].

This study had two aims: to assess trends in the overall and annual rates of CS and to investigate the relationship between CS and MMRs in Turkey with a subset of the Istanbul data from 2002 to 2013.

## Materials and Methods

This descriptive longitudinal study utilized information from large public datasets belonging to the Turkish Ministry of Health, Istanbul Health Administration, and National Maternal Mortality Study of Turkey. The Annual Clinical Reports for 2002–2013 were reviewed and analyzed. The study was approved by the local ethics committee of Fatih Sultan Mehmet Training and Research Hospital.

The eligibility criterion for inclusion in the study was maternal death, which was defined as follows: “the death of a woman while pregnant or within 42 days of termination of pregnancy, irrespective of duration and site of pregnancy, from any cause related to or aggravated by the pregnancy or its management, but not from accidental or incidental causes” [9]. According to the WHO, the term “direct maternal causes” refers to physiological and psychological conditions induced by the pregnancy itself. Indirect death can result from a previously existing disease or from a disease that develops during pregnancy and is not due to direct obstetric causes but is aggravated by the physiologic effects of pregnancy (including the puerperal period) [10].

The MMR refers to the number of maternal deaths per 100,000 live births from any cause related to or aggravated by pregnancy or its management, excluding accidental or incidental causes. In the present study, the direct obstetric causes of death included hemorrhage, infection/sepsis, and hypertensive disorders and embolism. Other physiological conditions caused directly by pregnancy included obstructed labor, unsafe abortion, ectopic pregnancy, and surgical and anesthetic problems during CS. The major indirect causes of death were cardiovascular conditions, cerebrovascular accidents, pulmonary system disorders, and gastrointestinal system disorders. Other indirect causes of death included diabetes, HIV/AIDS and anemia, all of which were preexisting or newly developed health problems that were unrelated to pregnancy, but were complicated or aggravated by it.

Antepartum hemorrhage is defined as genital bleeding during pregnancy from the 20th weeks to before delivery. Postpartum hemorrhage is defined as genital bleeding in the first 24 hours after delivery. Peripartum hemorrhage is defined as genital bleeding after 20 completed weeks of gestation to seven completed days after birth.

In this study, we investigated maternal deaths associated with hospital-based deliveries at ≥20 weeks of gestation that occurred from 1 January 2002 to 31 December 2013 in Istanbul and Turkey. The causes of maternal mortality, MMRs according to type of haemorrhage, CS rates during 2002–2013, correlation of CS rates with maternal deaths from peripartum hemorrhage, and comparison of CS rates during 2002–2007 and 2008–2013 were investigated according to the Istanbul data. Turkey data reflects both rural and urban data, however Istanbul data reflects mostly urban data.

## Statistical Analysis

The data were analyzed using IBM SPSS Statistics 22 (IBM SPSS, Turkey). The Shapiro–Wilk test was used to evaluate the normality of the data distribution. The Mann–Whitney U test was used to compare differences between two independent groups of data that were not normally distributed. Spearman’s rho correlation was used to evaluate the strength of the association between parameters. The chi-square test was used to detect group differences in categorical variables. A p-value of <0.05 was taken to indicate statistical significance.

## Results

A total of 2,047,381 pregnant women (≥20 weeks’ gestation) delivered 2,091,469 live infants from 2002 to 2013 in Istanbul, Turkey. From 2002 to 2013, there were 328 documented maternal deaths in Istanbul; 207 were due to causes directly related to pregnancy, and 121 were due to indirect causes. The main direct causes of death were hemorrhage (20%), hypertensive disorders (18.2%), embolism (10.3%), and sepsis/infection (8.5%). Eclampsia and preeclampsia accounted for 88.3% (53/60) of all hypertensive disorders. Most cases of infection were due to puerperal sepsis (21/28). The indirect causes of maternal mortality were cardiovascular disorders (9%), cerebrovascular accidents (7%), pulmonary system disorders (8%), gastrointestinal system disorders (4%), and other indirect causes (9%) (Table 1).

**Table 1:** The causes of maternal mortality in Istanbul.

	2002	2003	2004	2005	2006	2007	2008	2009	2010	2011	2012	2013	Total
**Direct Maternal Causes**													
Hemorrhage	7 (%23,3)	6 (%22,2)	6 (%25)	6 (%27,3)	5 (%20)	6 (%23,1)	5 (%16,7)	6 (%16,7)	5 (%20)	5 (%22,7)	4 (%11,8)	5 (%18,5)	66 (%20,1)
Hypertensive disorders	3 (%10)	4 (%14,8)	4 (%16,7)	5 (%22,7)	4 (%16)	6 (%23,1)	9 (%30)	4 (%11,1)	4 (%16)	4 (%18,2)	7 (%20,6)	6 (%22,2)	60 (%18,3)
Embolism	3 (%10)	2 (%7,4)	2 (%8,3)	2 (%9,1)	3 (%12)	2 (%7,7)	4 (%13,3)	5 (%13,9)	2 (%8)	2 (%9,1)	5 (%14,7)	2 (%7,4)	34 (%10,4)
Sepsis/Infection	3 (%10)	3 (%11,1)	2 (%8,3)	2 (%9,1)	3 (%12)	2 (%7,7)	3 (%10)	1 (%2,8)	2 (%8)	2 (%9,1)	3 (%8,8)	2 (%7,4)	28 (%8,5)
Other direct causes	3 (%10)	1 (%3,7)	2 (%8,3)	1 (%4,5)	2 (%8)	1 (%3,8)	1 (%3,3)	1 (%2,8)	1 (%4)	2 (%9,1)	2 (%5,9)	2 (%7,4)	19 (%5,8)
**Indirect Maternal Causes**													
Cardiovascular conditions	2 (%6,7)	3 (%11,1)	2 (%8,3)	2 (%9,1)	2 (%8)	2 (%7,7)	2 (%6,7)	4 (%11,1)	2 (%8)	2 (%9,1)	3 (%8,8)	3 (%11,1)	29 (%8,8)
Cerebrovascular accident	2 (%6,7)	1 (%3,7)	1 (%4,2)	1 (%4,5)	2 (%8)	1 (%3,8)	2 (%6,7)	3 (%8,3)	3 (%12)	1 (%4,5)	2 (%5,9)	2 (%7,4)	21 (%6,4)
Pulmonary system disorders	2 (%6,7)	3 (%11,1)	1 (%4,2)	1 (%4,5)	1 (%4)	2 (%7,7)	0 (%0)	8 (%22,2)	2 (%8)	1 (%4,5)	2 (%5,9)	2 (%7,4)	25 (%7,6)
Gastrointestinal system disorders	1 (%3,3)	1 (%3,7)	0 (%0)	1 (%4,5)	1 (%4)	2 (%7,7)	1 (%3,3)	2 (%5,6)	2 (%8)	1 (%4,5)	2 (%5,9)	1 (%3,7)	15 (%4,6)
Other indirect causes	4 (%13,3)	3 (%11,1)	4 (%16,7)	1 (%4,5)	2 (%8)	2 (%7,7)	3 (%10)	2 (%5,6)	2 (%8)	2 (%9,1)	4 (%11,8)	2 (%7,4)	31 (%9,5)

A total of 23 patients died of ante-intrapartum haemorrhage and 13 died of postpartum hemorrhage from 2007 to 2013 in Istanbul (Table 2). There were no significant differences in ante-intrapartum haemorrhage bleeding (p > 0.05) or postpartum hemorrhage (p > 0.05) from 2007 to 2013 (Table 2).

**Table 2:** Maternal mortality ratio (MMR) according to type of bleeding.

Year	Ante-intrapartum hemorrhage	Postpartum hemorrhage
**2007**	4	2
**2008**	3	2
**2009**	4	2
**2010**	3	2
**2011**	4	1
**2012**	2	2
**2013**	3	2
**Total**	23	13

In total, 960,921 CS deliveries were performed in Istanbulfrom 2002 to 2013, during which time the CS rate increased from 23.0% in 2002 to 55.2% in 2013. Overall, the average annual CS delivery rate was 46.4% in Istanbul and 36.6% in Turkey (Table 3). There was a significant increase in the CS rates in Istanbul and Turkey from 2008 to 2013 relative to those from 2002 to 2007 (p = 0.004) (Table 4).

**Table 3:** Cesarean section (CS) rates during 2002–2013.

Year	Turkey	Istanbul
**2002**	23	21
**2003**	31.5	21
**2004**	39.5	25
**2005**	43.1	29
**2006**	46.9	32
**2007**	47.1	36
**2008**	49.1	37
**2009**	52.5	43
**2010**	56.2	46
**2011**	55.7	47
**2012**	56.4	51
**2013**	55.2	51

**Table 4:** Comparison of CS rates during 2002–2007 and that during 2008–2013.

	2002–2007	2008–2013	P
	Median ± SS	Median ± SS	
**Turkey**	38.52 ± 9.55 (41.3)	54.18 ± 2.86 (55.5)	0.004**
**Istanbul**	27.33 ± 6.09 (27)	45.83 ± 5.30 (46.5)	0.004**

Mann–Whitney U-test

**p < 0.01

The overall MMR from 2002 to 2013 was 31.6 in Turkey and 16.3 in Istanbul. There was a significant decrease in the MMR from 2002 to 2013 in Turkey (p < 0.05) (Table 5).

**Table 5:** MMR distribution (1/100000).

Year	Turkey	Istanbul
**2002**	64	22
**2003**	61	19
**2004**	50.2	15.9
**2005**	39.2	12.6
**2006**	28.5	14.1
**2007**	21.2	14.1
**2008**	19.4	17.2
**2009**	18.4	20.8
**2010**	16.4	13.5
**2011**	15.5	10.6
**2012**	15.4	17.3
**2013**	19.9	18.2

**Fig 1:**
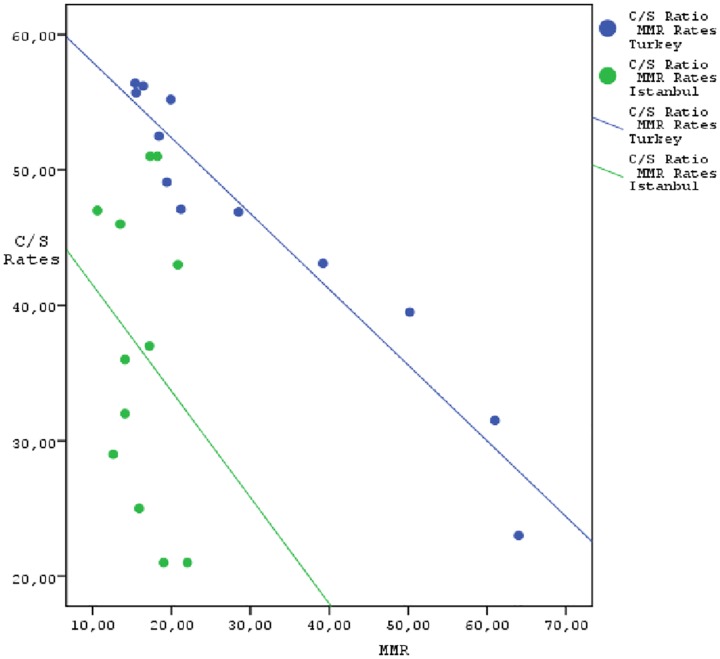
The correlation of cesarean rates and maternal mortality rates between 2002–2013 in Istanbul and Turkey.

There was a statistically significant and inverse relationship (97.2%) between the maternal mortality ratios and CS rate from 2002 to 2013 in Turkey (p = 0.001). However, no significant relationship was detected between the maternal mortality ratios and CS rate from 2002 to 2013 in Istanbul (p > 0.05) (Table 6) (Fig 1).

**Table 6:** The correlation of maternal mortality ratios and caserean rates between 2002–2013.

Maternal Mortality Ratio (1/100000)	Ceserean Section Rates			
	Turkey		Istanbul	
	R	p	r	p
**Turkey**	-0,972	0,001**	-	-
**Istanbul**	-	-	-0,204	0,525

Spearman’s Rho Correlation

**p<0.01

There was a significant inverse correlation (66.3%) between the CS rate and peripartumhemorrhage in Turkey (p = 0.019) and there was a significant inverse correlation (66.5%) between the CS rate and peripartumhemorrhage(p = 0.018) in Istanbul between 2007 to 2013 (Table 7).

**Table 7:** Correlation of cesarean rates and maternal deaths from peripartum hemorrhage during 2007–2013.

Deaths caused by peripartum hemorrhage	Cesarean section rates			
	Turkey		Istanbul	
	r	p	r	p
**Third-trimester bleeding**	-0.449	0.312	-0.415	0.354
**Postpartum hemorrhage**	0.449	0.312	0.415	0.354
**Total**	-0.663	0.019*	-0.665	0.018*

Spearman’s Rho Correlation

*p < 0.05

All Turkey data include the Istanbul data.

## Discussion

This study evaluated the outcomes of CS and the MMR in a wide representative sample of patients spanning a long period of time. This study was limited by the fact that the data reflected only hospital-based deliveries and that there was insufficient information regarding deaths that occurred in the home and those that were not recorded officially. Additionally, there was no standardized reporting system among hospitals, making it difficult to compare the data.

This study demonstrates a clear increase in the CS rate from 2002 to 2013. During this same time period, the MMR decreased significantly, especially in the earlier years. We determined that the prominent causes of maternal mortality were postpartum hemorrhage and hypertensive disorders. Thromboembolism was the third most frequent cause, which is not the case in developed countries. The 2009 H1N1 influenza viruspandemic in Turkey, which was responsible for seven cases of maternal mortality, increased the number of deaths associated with respiratory infection that year.

It was difficult to find trustworthy data on ante-intrapartum haemorrhage and postpartum hemorrhage, for all years. Therefore, we considered and evaluated data from 2007 to 2013 only, and we found no significant changes in these parameters during that time period.

This study showed no significant increase in the incidence of postpartum hemorrhage and related maternal deaths in Istanbul and Turkey commensurate with the substantial increase in the CS rate from 2002 to 2013. However, negative impacts associated with the increased application of CS are expected during the next few decades because the increase in the incidence of CS in Turkey is a relatively recent phenomenon.

The health policy in Turkey changed significantly after 2003, when the Ministry of Health achieved unification and standardization of all hospitals. This change allowed all patients to apply for government-funded treatment in both public and private hospitals, and the subsequent decrease in the MMR was probably due to new improvements in the general standards of the health care system and intensive care units (ICUs) in Turkey.

The neonatal mortality rate decreased from 45 per 1000 live births in 2002 to 23 per 1000 live births in 2013 [11]. Additionally, the life expectancy of women increased from 71 to 75 years in the same time period [12]). When we compared the overall data in Turkey, the CS rate and MMR showed a significant negative correlation from 2002 to 2013. However, there were no significant correlations when we considered the data from Istanbul during this period. We assume that this difference was due to the fact that Istanbul has an urban population with higher health standards than those of the both rural and urban population of Turkey.

With the improvements in health care facilities during the past decade, patients can easily access emergency wards and ICUs. Almost 80% of all maternal deaths could be prevented if women had immediate access to basic maternity and health care services; 45% of postpartum deaths occur within 24 hours [13,14]. In emergency situations, the new health insurance system in Turkey covers every patient without any restrictions, including ICU admission expenses. Furthermore, preventive medicine facilities and primary care services are supported, and the family medicine system has been expanded to every neighborhood. A performance reward system has been established for health care government paid medical workers; for example, doctors earn more money if they examine more patients.

Before 2003, the number of pregnant women who gave birth in private hospitals was relatively low for economic reasons. Since 2003, the number of deliveries in private hospitals has increased commensurate with the substantial increase in the number of such institutions. The state supports the private sector; therefore, private doctor-assisted deliveries have increased [15]. However, this system increased the CS rate to as high as 90% in some private hospitals in Istanbul. Also particularly over the past 10 years, fear of litigation in Turkey has led physicians to practice defensive medicine; the increase in private health care institutions represents an additional factor underlying the higher CS rate [16].

Turkey was the first country to make elective CS punishable by law in an effort to decrease the performance of unnecessary primary CS [17]. “Once a CS, always a CS” is a phrase overused by doctors reluctant to attempt vaginal deliveries. Case studies demonstrate the economic and availability concerns of physicians influence the likelihood that CS delivery will be performed [18]. A Cochrane Review reported no evidence from randomized controlled trials upon which to base practice recommendations regarding planned CS for nonmedical reasons at term [19].

Similar to the situation in some developed countries, medical malpractice cases against obstetricians have increased in Turkey and now represent half of all forensic medicine cases [20]. Therefore, malpractice insurance (paid for by doctors) is obligatory, and obstetricians thus feel obliged to practice defensive medicine and take no risks during the course of delivery.

Maternal death and disability from hemorrhage, infection, and obstructed labor may be averted by timely CS. Most LICs have CS rates less than those recommended by the WHO. Without access to timely CS, it is unlikely that the MMRs in LICs will be further reduced. The current MMRs in LICs should be decreased by raising the CS rates in these countries to the WHO-recommended levels (10%–15%) [21].

A recent study demonstrated that a national CS rate of up to approximately 19 per 100 live births is associated with lower maternalor neonatalmortalityamong WHO member states [22]. It can be assumed that the previously recommended national targetratesfor CS deliveries may be too low. Higher CS delivery rates were not correlated with maternal or neonatal mortality at a country level in that study. A sensitivity analysis including only 76 countries demonstrated that delivery rates of >6.9 to 20.1 per 100 live births were inversely correlated with the MMR [22].

The present study suggests that improved health care facilities also improve the MMR. Future studies should evaluate the relationship between the MMR and CS rates while controlling for other confounding factors such as thromboembolism, anesthesia, and placenta accreta. Future studies should also include home-based deliveries using standardized reporting. In the absence of trial data, there is an urgent need for a systematic review of observational studies and a synthesis of qualitative data to better assess the short- and long-term effects of increased CS rates and maternal mortality.

To decrease CS rates, vaginal deliveryshould beencouraged and excessive legal pressure concerning deliveries must be alleviated. The present study provides important information that might be useful for other low-and middle income countries with demographic and economic similarities.

## Conclusions

This study demonstrated an inverse correlation between increased CS rates and MMRs during the past decade in Turkey. Although cesarean rates increase excessively, it appears that improved health care facilities have a positive effect on MMRs in Turkey.
